# Cohort Profile Update: The Swiss Childhood Cancer Survivor Cohort

**DOI:** 10.1093/ije/dyag089

**Published:** 2026-06-15

**Authors:** Grit Sommer, Christina Schindera, Carina Nigg, Nicolas Waespe, Nicolas X Von Der Weid, Fabiën N Belle, Claudia E Kuehni

**Affiliations:** Childhood Cancer Research Group, Institute of Social and Preventive Medicine, University of Bern, 3012 Bern, Switzerland; Childhood Cancer Research Group, Institute of Social and Preventive Medicine, University of Bern, 3012 Bern, Switzerland; Division of Pediatric Oncology/Hematology, University Children’s Hospital Basel, University of Basel, 4031 Basel, Switzerland; Childhood Cancer Research Group, Institute of Social and Preventive Medicine, University of Bern, 3012 Bern, Switzerland; Childhood Cancer Research Group, Institute of Social and Preventive Medicine, University of Bern, 3012 Bern, Switzerland; Division of Pediatric Hematology and Oncology, Department of Pediatrics, Inselspital, Bern University Hospital, University of Bern, 3010 Bern, Switzerland; CANSEARCH Research Platform for Pediatric Oncology and Hematology, Department of Pediatrics, Gynecology and Obstetrics, Faculty of Medicine, University of Geneva, 1211 Geneva, Switzerland; Division of Pediatric Oncology/Hematology, University Children’s Hospital Basel, University of Basel, 4031 Basel, Switzerland; Childhood Cancer Research Group, Institute of Social and Preventive Medicine, University of Bern, 3012 Bern, Switzerland; Childhood Cancer Research Group, Institute of Social and Preventive Medicine, University of Bern, 3012 Bern, Switzerland; Division of Pediatric Hematology and Oncology, Department of Pediatrics, Inselspital, Bern University Hospital, University of Bern, 3010 Bern, Switzerland

**Keywords:** childhood cancer, cohort study, late effects, survivor, Switzerland, Europe

Key Features

- The Swiss Childhood Cancer Survivor Study is a national, lifelong follow-up of individuals diagnosed with cancer before age 20 years.
- Since its launch in 2007, the study has continuously enrolled new 5-year survivors, expanded data collection through questionnaires, and broadened its research scope.
- As of 31 July 2025, 4135 survivors or their guardians had completed the baseline questionnaire: 1291 when aged 5–15 years, 665 when aged 16–19 years, and 2179 when aged >20 years; 1805 had also completed at least one follow-up questionnaire. In total, 1175 siblings participated and serve as a comparison group.
- Recent developments include data linkages with hospital-based electronic medical records via the Swiss Personalized Health Network, follow-up questionnaires, standardized examinations targeting cardiac, pulmonary, and otologic late effects, and germ-line DNA samples.
- For new collaborations or enquiries about data sharing, please contact sccss.ispm@unibe.ch or visit the study website at www.swiss-ccss.ch.

## The original cohort

Although childhood cancers are rare—affecting <1 in 2000 individuals—they remain the leading cause of disease-related death among children and adolescents aged <20 years in high-income countries [1]. Thanks to advances in treatment, survival rates have improved from ∼20% in the 1940s to almost 90% today in high-income countries [2, 3]. However, many survivors face long-term physical and psychosocial complications from the cancer itself or its intensive treatment [4]. As with other rare diseases, centralized data collection is essential for robust, generalizable evidence. In response, the Swiss Pediatric Oncology Group (SPOG) established a national childhood cancer registry for Switzerland in 1976. Building on this, the Swiss Childhood Cancer Survivor Study (SCCSS) was launched in 2007 to provide lifelong follow-up of all individuals registered since 1976 who survived for ≥5 years post-diagnosis. The initial cohort profile, published in 2011, described the setup, the study population as of December 2010, and baseline survey data from 1605 survivors [5]. The study is registered at clinicaltrials.gov (NCT03297034).

## What is the reason for the new focus?

Since its inception, the SCCSS has evolved through multiple phases. Data collection has expanded, the legal and research infrastructure in Switzerland has changed, and the study has broadened its scope in response to emerging scientific questions.

### Ongoing enrolment

**Figure 1:**
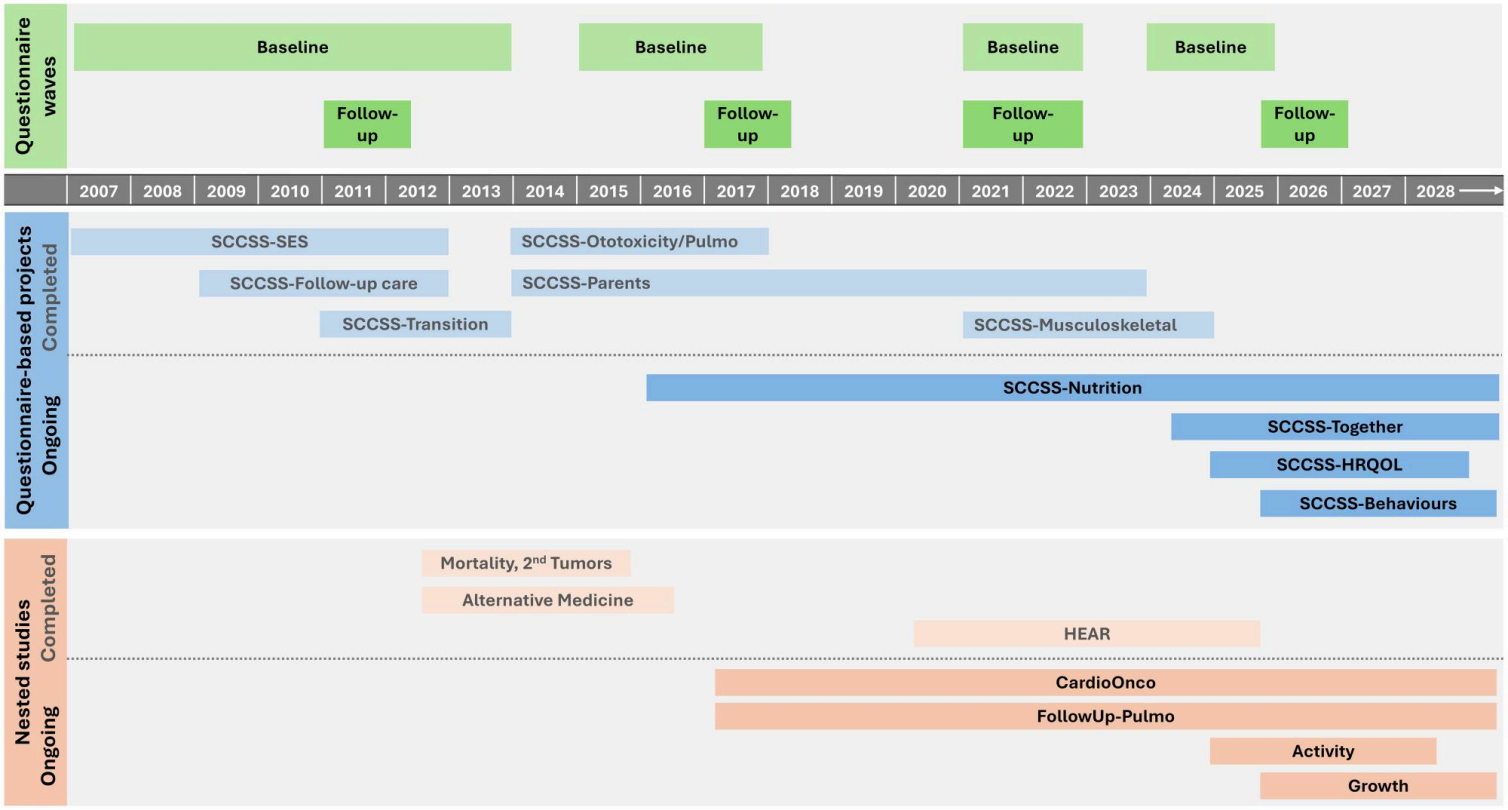
Timeline of the SCCSS with questionnaire waves, questionnaire-based projects, and nested studies. Questionnaire waves are shown above the time bar, projects based on questionnaires or records are shown below the time bar, and nested studies are shown at the bottom of the figure. Ongoing studies (July 2025) are shown in darker colors and font; completed studies are shown in lighter shades. HRQoL, health-related quality of life. Questionnaire-based projects: SCCSS-SES: socioeconomic status and associations with childhood cancer incidence and survival, 2007–12; SCCSS-Follow-up care: follow-up care after childhood and young adult cancer, 2009–12; SCCSS-Transition: effectiveness of transition to adult care after childhood cancer, 2011–13; SCCSS-Ototoxicity/Pulmo: ototoxicity, pulmonary outcomes, and quality of life in childhood cancer survivors, 2014–17; SCCSS-Parents: parents of long-term childhood cancer survivors, 2014–23; SCCSS-Musculoskeletal: musculoskeletal outcomes and fatigue in childhood cancer survivors, 2021–24; SCCSS-Nutrition: dietary habits, nutrition, and risk of late effects after childhood cancer, since 2016; SCCSS-Together: a participatory in-depth study involving survivors of childhood cancer, since 2024; SCCSS-HRQoL: trends of health-related quality of life after childhood cancer, since 2024; SCCSS-Behaviours: time trends and clusters of health behaviors, since 2025; Nested studies: Mortality, 2nd Tumors: mortality and second primary cancers after cancer in childhood and adolescence, 2012–15; Alternative Medicine: use of complementary and alternative medicine in children with cancer, 2012–16; HEAR: improving access to screening for hearing loss after childhood cancer, 2020–25; CardioOnco: cardiovascular disease after childhood cancer: diagnosing early-stage disease, since 2017; FollowUp-Pulmo: pulmonary dysfunction after childhood cancer: diagnosing early-stage disease, since 2017; Activity: accelerometer-assessed physical activity, sedentary behavior, and sleep, since 2024; Growth: trajectories of growth and weight after childhood cancer from birth until adulthood, since 2025.

The SCCSS began recruitment in 2007, initially targeting adult childhood cancer survivors (aged ≥20 years), followed by adolescent survivors (16–19 years) and younger survivors (5–15 years), for whom parents completed the questionnaire. New 5-year survivors have been invited in regular waves, leading to a substantial increase in the numbers of participants—from 1605 individuals in 2011 to 4135 by 31 July 2025 (Table 1 and Fig. 1**)**.

**Table 1:** The SCCSS in 2025: completed questionnaires and response rates by age group for the different baseline follow-up survey waves.

Participants (response rate)				
	Adults (aged ≥20 years at survey) [*n* (%)]	Adolescents (aged 16–19 years at survey) [*n* (%)]	Children (aged 5–15 years at survey) [*n* (%)]	Total [*n* (%)]
**Baseline surveys**				
Wave 2007–13 (diagnosed 1976–2005)	1596 (71)	464 (73)	467 (77)	2527 (73)
Wave 2015–17 (diagnosed 2006–2010)	238 (55)	80 (61)	334 (73)	652 (64)
Wave 2021–22 (diagnosed 2011–2015)	240 (56)	65 (50)	284 (60)	589 (57)
Wave 2024–2025 (diagnosed 2016–2019)	105 (41)	56 (42)	206 (50)	367 (47)
**Total as of 31 July 2025**	**2179 (65)**	**665 (65)**	**1291 (66)**	**4135 (65)**
**Follow-up surveys**				
Follow-up 2011 (adult survivors with baseline questionnaire)	511 (51)	NA	NA	511 (51)
Follow-up 2017 (adult survivors with baseline questionnaire)	919 (57)	NA	NA	919 (57)
Follow-up 2021/22 (adolescent and adult survivors with baseline questionnaire, not in Follow-up 2011)	264 (44)	111 (54)	NA	375 (46)
Follow-up 2026 (adult survivors with baseline or follow-up questionnaire ≥5 years earlier)	Ongoing	NA	NA	Ongoing
**Total as of 31 July 2025**	**1694 (53)**	**111 (54)**	**NA**	**1805 (53)**

NA, not applicable.

### Follow-up questionnaires

For longitudinal analysis, follow-up questionnaires were introduced for participants with a baseline questionnaire to track long-term outcomes across the life course. To date, 1805 survivors have completed a follow-up questionnaire. A new follow-up wave is currently ongoing (2025/2026).

### Legal developments

In 2020, Switzerland implemented a national cancer-registration law, mandating cancer reporting and providing structural funding for the Childhood Cancer Registry (ChCR, www.childhoodcancerregistry.ch). This law formalized a separation between the registry (focused on monitoring) and the SCCSS (focused on research), necessitating adaptations in cohort logistics and ethics procedures.

### New research infrastructure within Switzerland

The launch of the Swiss Personalized Health Network (SPHN, www.sphn.ch) in 2017 has offered new opportunities for the SCCSS. The SPHN creates the framework that makes health-related data accessible, interoperable, and usable for personalized medicine and research in Switzerland. The SPHN supports linkage with hospital-based clinical data and enhances the integration of real-world medical information into research [6].

### Expansion of study scope

Beyond questionnaire-based projects, the SCCSS initiated nested studies on cardiac, pulmonary, and ototoxic late effects, physical activity, and growth. Nested studies involve standardized clinical assessments for deeper phenotyping of long-term complications. In 2019, nationwide germ-line DNA collection started in subgroups and is ongoing for all survivors and newly diagnosed patients.

### Participatory research

The SCCSS has strengthened its commitment to participatory research by actively involving survivors, families, and parent support organizations. In addition to ongoing engagement with advocacy groups, we also established a Family Advisory Group (SCCSS-Gemeinsam). It proposes new research questions, reviews study proposals, provides feedback on questionnaires, helps in interpreting findings, and ensures that results are accessible and relevant. To promote transparency and inclusion, all scientific publications are now accompanied by plain-language summaries for non-academic audiences (www.swiss-ccss.ch).

## What will be the new areas of research?

The SCCSS has expanded its scope substantially. New projects build on both questionnaires and nested studies, with an increasing focus on longitudinal follow-up, objectively measured outcomes, and integration with genetic and clinical data (see Fig. 1).

### Questionnaire-based SCCSS projects

- With almost a decade of data collection across multiple enrolment waves, the SCCSS can now investigate secular trends. For example, we analyse changes in hearing impairment, physical activity, sun-protection behaviors, body weight, and smoking in the context of modified cancer therapies and evolving public health measures such as tobacco legislation.
- SCCSS-Nutrition investigates dietary intake and quality in adult survivors (aged ≥18 years) via multiple dietary assessment tools, e.g. food frequency questionnaires and urine samples [7]. Data collection is ongoing, with 802 survivors having participated so far.
- Longitudinal analyses explore how outcomes evolve over time within individuals. Ongoing studies assess changes in health-related quality of life (HRQoL), growth, and overweight by using repeated questionnaire data. For selected outcomes such as height and weight, repeated measures from hospital electronic records integrated via the SPHN complement self-reported data.

### Nested studies

These go beyond self-reported outcomes, by collecting standardized clinical data:

- “CardioOnco” is conducted in five Swiss hospitals and offers standardized cardiac evaluations—including advanced echocardiography and cardiopulmonary exercise testing—to adult survivors (aged ≥18 years) [8]. To date, 590 survivors have completed baseline assessments and 250 have longitudinal follow-up.
- “Pulmo” is active in three pediatric hospitals and offers standardized lung-function tests to survivors <18 years of age [9]; 251 have participated so far.
- “HEAR” offered a community-based hearing test through partnerships with hearing-aid providers across Switzerland [10]. Survivors were invited for a free pure-tone audiogram; 476 consented and 319 completed the audiogram in this nonclinical setting.
- “Activity” investigates physical activity, sedentary behavior, and sleep over 1 week by using thigh-worn accelerometers. All survivors in the SCCSS are eligible to participate. Data collection is ongoing, with 93 survivors having participated so far.
- “Growth” investigates underweight, overweight, and linear growth in childhood cancer patients and survivors. We use height and weight measurements routinely collected by all treating hospitals and link these data to the SCCSS for post-treatment self-reported heights and weights. Data linkage is ongoing.

### Genetic studies

- Germ-line DNA is collected via postal saliva kits and associated clinics, as part of the BISKIDS (Germline DNA Biobank Switzerland for Childhood Cancer and Blood Disorders) initiative [11]. In the GECCOS (Genetic Risks for Childhood Cancer Complications Switzerland) project, whole-exome and whole-genome sequencing identify genetic variants associated with long-term complications of childhood cancer and its treatment [12].

These new research areas substantially enhance the SCCSS’s capacity to study the lifelong trajectory of childhood cancer survivors, combining self-reported, clinical, genetic, and behavioral data in a comprehensive epidemiological framework.

## Who is in the cohort?

The source population for the SCCSS is the ChCR—a national, population-based registry, founded in 1976 by the SPOG, which moved in 2003 to the Institute of Social and Preventive Medicine (ISPM) at the University of Bern. In 2020, with the implementation of the cancer-registration law in Switzerland, the reporting of neoplasms became mandatory and the registry was transformed into a federally funded entity, mandated to the University of Bern (www.childhoodcancerregistry.ch). It includes all children and adolescents diagnosed before age 20 years in Switzerland with leukemia, lymphoma, central nervous system tumors, other solid malignancies, and Langerhans cell histiocytosis, classified according to the International Classification of Childhood Cancer, third edition (ICCC-3) [13].

### Follow-up by SCCSS questionnaire surveys

As of 31 July 2025, the ChCR included 13 963 patients and 8036 were still alive. The SCCSS is a dynamic cohort, into which new participants are invited in waves as they reach eligibility (5-year survival) (Fig. 1). In 2007, we invited all 5-year survivors to complete a baseline questionnaire, tailored to their current age: children (5–15 years of age), adolescents (16–19 years of age), or adults (≥20 years of age). Initially, most participants were adult long-term survivors. Since then, the SCCSS has invited new 5-year survivors in waves to join the cohort (Tables 1 and 2). Currently, 4135 have completed the baseline questionnaire and 1805 have completed two questionnaires (Table 1); 1175 siblings took part as a comparison group (Supplementary Table 1).

**Table 2:** Characteristics of participants in the SCCSS questionnaire surveys in 2025.

	All eligible survivors^a^ (*n* = 6912) [*n* (%)]	SCCSS responders^b^ (*n* = 4135) [*n* (%)]
**Socio-demographic characteristics**		
Sex		
Male	3865 (56)	2221 (54)
Female	3044 (44)	1914 (46)
Attained age at study (years)		
5–9	582 (8)	332 (8)
10–15	1505 (22)	961 (23)
16–19	1151 (17)	706 (17)
≥20	3674 (53)	2136 (52)
Language region of Switzerland		
German	4552 (68)	2892 (70)
French	1942 (29)	1093 (26)
Italian	225 (3)	150 (4)
**Clinical characteristics**		
Diagnosis, ICCC-3		
I. Leukemia	1982 (29)	1334 (32)
II. Lymphomas	1258 (18)	703 (17)
III. CNS tumors	1179 (17)	681 (17)
IV. Neuroblastoma	321 (5)	205 (5)
V. Retinoblastoma	176 (3)	115 (3)
VI. Renal tumors	335 (5)	223 (5)
VII. Hepatic tumors	51 (1)	31 (1)
VIII. Bone tumors	307 (4)	165 (4)
IX. Soft tissue sarcomas	436 (6)	258 (6)
X. Germ cell tumors	441 (6)	195 (5)
XI. Other malignant epithelial neoplasms	195 (3)	85 (2)
XII. Other and unspecified malignant neoplasms	16 (<1)	6 (<1)
Langerhans cell histiocytosis	212 (3)	134 (3)
Age at diagnosis (years)		
<5	2472 (36)	1646 (40)
5–9	1411 (20)	955 (23)
10–15	1464 (21)	879 (21)
16–20	1562 (23)	655 (16)
Time since diagnosis (years)		
5–10	3976 (58)	2114 (51)
11–15	1144 (17)	725 (18)
16–20	632 (9)	457 (11)
≥20	1157 (17)	839 (20)
Year of diagnosis		
≤1979	246 (4)	170 (4)
1980–89	1001 (15)	703 (17)
1990–99	1612 (23)	1107 (27)
2000–09	1866 (27)	1172 (28)
2010–19	2184 (32)	983 (24)

Percentages are based upon available data for each variable. ^a^Includes all survivors eligible at the time of study (i.e. when contacting them). ^b^Includes all survivors who responded to the baseline questionnaire.

CNS, central nervous system.

### Follow-up via data linkages for survival, causes of death, and second primary neoplasms

Follow-up for second primary neoplasms and for causes of death can be performed for the entire cohort (*N* = 13 963) through linkages with cantonal cancer registries that collect tumors for people aged ≥20 years and linkages with vital statistics and cause-of-death statistics at the Swiss Federal Statistical Office, minimizing selection bias.

Follow-up via interoperable data contained in the clinical data warehouses of the treating hospitals is now possible for those treated in the five University Hospitals at Basel, Bern, Geneva, Lausanne, and Zurich, and partially for those treated in the four Cantonal Hospitals at Aarau, St. Gallen, Lucerne, and Bellinzona.

Germ-line DNA is being collected (response rate of ∼50%) and analysed in batches. As of March 2025, DNA samples were available for 694 survivors and whole-genome sequencing data for 221.

### Participants in nested studies

Table 3 describes the participants in nested studies on cardiac, pulmonary, and hearing outcomes, physical activity, and dietary assessments.

**Table 3:** Childhood cancer survivors participating in ongoing projects and nested studies with clinical data collection within the SCCSS.

	Inclusion criteria	*N* with baseline assessment (% response rate)	Median age (range) at baseline in years	*N* with ≥1 follow-up assessment^a^
CardioOnco (since 2016)	Diagnosed with childhood cancer Registered in the ChCR Survived ≥5 years since diagnosis Aged ≥18 years at study Treated with any chemotherapy and/or cardiac radiation Treated at the University Children’s Hospitals in Bern, Basel, Luzern, St. Gallen, or Geneva	590 (38)	31 (24–39)	250
Pulmo (since 2017)	Diagnosed with childhood cancer Aged 6–20 years at study Survived ≥1 year since diagnosis Treatment completed Treatment with any chemotherapy, radiation to thorax, spine or abdomen, thoracic surgery, or HSCT In regular pediatric hemato-oncological follow-up care in the University Children’s Hospitals in Bern, Basel, or Geneva	251 (90)	14 (11–17)	∼80 planned
HEAR (2022‒25)	Diagnosed with childhood cancer Registered in the ChCR Survived ≥2 years since diagnosis Aged ≥18 years at study Treated with any chemotherapy or radiation to the head, neck, or spine of any dose	319 (20)	33 (18–59)	NA
Activity (since 2024)	Diagnosed with childhood cancer Registered in the ChCR Aged <20 years at diagnosis Survived ≥5 years since diagnosis Completed an SCCSS baseline questionnaire Participated in accelerometer study	108 (29)	14 (10–21)	NA
SCCSS-Nutrition (since 2016)	Diagnosed with childhood cancer Registered in the ChCR Aged ≥18 years at study Survived ≥5 years since diagnosis Participated in an SCCSS baseline questionnaire Not pregnant/lactating at time of study No missing or implausible dietary intake information reported in the FFQ	802 (54)	35 (21–59)	NA

a 2–5 years after baseline assessment.

FFQ, food frequency questionnaire; HSCT, hematopoietic stem-cell transplantation; NA, not applicable.

### Current age of cohort members

By 31 July 2025, the cohort had included participants aged between 6 and 69 years, with a median age of 33 years (Supplementary Table 2).

## What has been measured?

**Figure 2:**
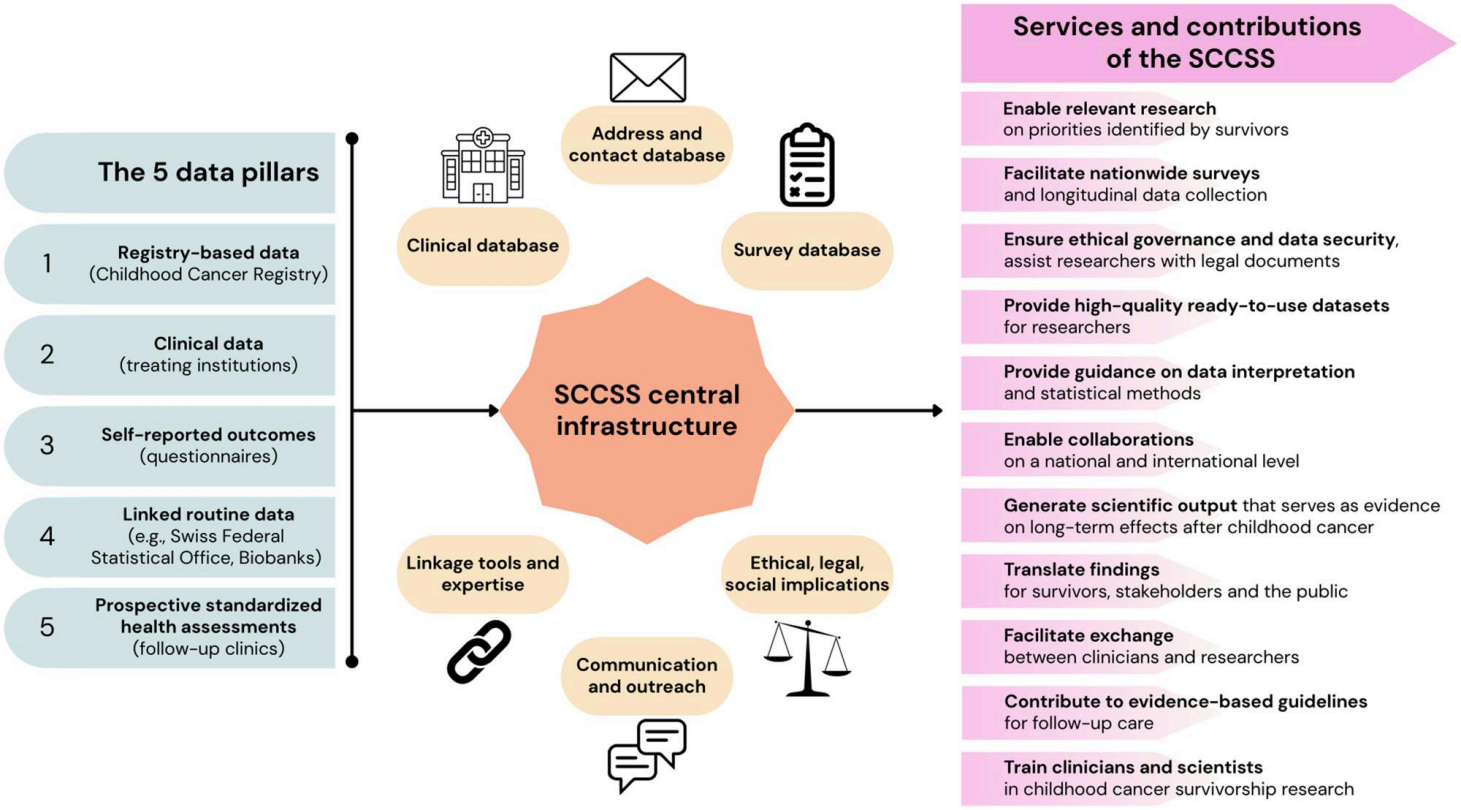
Schematic overview of the SCCSS.

The SCCSS has five distinct data pillars (Table 4 and Fig. 2). First, registry-based data provide basic and supplementary data on cancer diagnosis and treatment. Second, clinical data are extracted from medical records in the respective hospitals. Third, self-reported outcomes from postal and online questionnaires include personal information, HRQoL, medical care, medication use, healthcare utilization, somatic and mental health, fertility and pregnancy, lifestyle and health behaviors, education, and socioeconomic status. Questions were originally taken from the North American Childhood Cancer Survivor Study (https://ccss.stjude.org) to enable cross-national comparison of health outcomes. Where country-specific comparison was important, questions were sourced from Swiss population health questionnaires such as the Swiss Health Survey and Swiss Census including household income, lifestyle, and health behaviors. A distinct feature of the SCCSS is the inclusion of both adults (aged ≥20 years) and young survivors, starting from 5 years post-diagnosis. Questionnaires are provided in the three national languages of German, French, and Italian. The fourth data source is linked routine data from the Swiss Federal Statistical Office, cantonal cancer registries, the SPHN, and biobanks. Fifth, nested studies prospectively measure health outcomes by using objective testing.

**Table 4:** Data pillars for the SCCSS and the type of data collected from each pillar.

Time frame	Sample addressed	Description/measurements
**1. Registry-based datafrom the Swiss ChCR**		
Since 1976	All children in the ChCR	Basic data: diagnostic information, metastases, and relapses. Information about initial treatment: type and objective of treatment, facts on treatment decision, treatment start Supplementary data: information on further treatments: type and objective of treatment, facts on treatment decision, treatment start, and result. Cancer predispositions, previous diseases, and comorbidities Full data structure is available at the website of the NACR: www.nkrs.ch
**2. Clinical data from medical records**		
Since 1976	All children in the ChCR	Detailed therapy, complications, disease trajectory collected from medical records, diagnostic test results (e.g. lung function tests, audiograms)
**3. Self-reported outcomes from questionnaires**		
Since 2007	Children diagnosed since 1976 who survived ≥5 years	Postal and online questionnaires including personal information, HRQoL, medical care, medication use, healthcare utilization, somatic and mental health, fertility and pregnancy, lifestyle, health behaviors, education, socioeconomic status
**4. Linked routine data from cancer registries and Federal Statistical Office**		
Since 2010	All children in the ChCR	Cantonal Cancer Registries and NACR: assessment of secondary malignancies: details of tumor diagnosis, date of diagnosis, causes of death
Since 2005	All children in the ChCR	Updates via communities: vital status, date of death, address history
Since 2005	All children in the ChCR	Linkage with datasets from the FSO and SNC: mortality, migration, birth statistics (STATPOP, BEVNAT) Swiss Census datasets hospital episode statistics (“Medizinische Statistik der Krankenhäuser”) annual structural surveys SNC: environmental exposures and socioeconomic situation
Since 2017	All children in the ChCR	SPHN: routine clinical data captured in the data warehouses of treating hospitals in a standardized and interoperable format. Information on available data concepts, structure, and semantics can be found in the SPHN Metadata Catalog
Since 2019	All children in the ChCR	Biobanking: BISKIDS is a nationwide germ-line DNA biobank collecting germ-line DNA and sequencing data. The SPHO Biobank Network is a multisite network cooperating with the Departments of Pathology and University Hospitals that links decentralized tumor biobanks and enables access to tumor samples Biolink: allows linkage of the SCCSS database and ChCR with BISKIDS (Geneva) and SPHO Biobank Network (Zurich) in a secured, privacy-preserving way, to facilitate and support translational research
**5. Prospective standardized health assessments (nested studies)**		
Since 2017	Study-specific sub-cohorts	Prospective data on late effects and health status, including: pulmonary function assessments: spirometry, body plethysmography, DLCO, N_2_MBW, FeNO, respiratory health questionnaire cardiac function assessments: conventional echocardiography (2D and 3D), 2D speckle tracking echocardiography, anthropometry, blood pressure, cardiopulmonary exercise testing, physical examination (auscultation of heart and lungs, palpation of pulses, signs of heart failure), 1-minute sit-to-stand test, personal history, questionnaires on quality of life, fatigue, and health behaviors hearing assessment: pure-tone audiometry from 125 to 8000 Hz and, for participants with hearing loss of >25 decibels, bone conduction from 250 to 4000 Hz dietary assessments: urine samples and food frequency questionnaires physical behavior assessment: physical activity, sedentary behavior, and sleep via thigh-worn accelerometry (activPAL 4) for 1 week

DLCO, diffusion capacity of the lung for carbon monoxide; N_2_MBW, nitrogen multiple breath washout test; NACR, National Agency for Cancer Registration; FeNO, fractional exhaled nitric oxide (measured in childhood cancer survivors with symptoms suggestive of asthma, e.g. wheeze, dyspnea, cough, or signs of obstruction in spirometry); FSO, Federal Statistical Office; Hz, Hertz; SNC, Swiss National Cohort; SPHO, Swiss Pediatric Hematology Oncology.

## What has it found? Key findings and publications

Until now, the SCCSS has resulted in >150 publications on a variety of topics, including psychosocial and mental outcomes, health behaviors such as physical activity, alcohol drinking, and smoking, and medical follow-up care [5]. Nested studies with dedicated funding have enabled the investigation of somatic late effects. Exemplary findings include:

- Cardiac late effects: We found that survivors of pediatric acute lymphoblastic leukemia had a 2-fold increased risk for cardiovascular disease compared with siblings, with the highest risk after anthracycline exposure, chest radiation, and hematopoietic stem-cell transplantation [14]. The hospital-based CardioOnco Study reported on cancer-related fatigue and physical strength and endurance [15, 16]; cardiac analyses are ongoing [17].
- Pulmonary late effects: We found increased rates of pneumonia and chest-wall abnormalities among survivors, with a cumulative incidence of any pulmonary disease of 21% 35 years post-treatment [18]. We investigated lung function [19] and formulated evidence-based recommendations for pulmonary follow-up as part of the International Late Effects of Childhood Cancer Guideline Harmonization Group [20]. The Pulmo Study published on exercise-induced respiratory problems and lung-function data [9, 21, 22].
- Hearing: We found that three out of four survivors treated with platinum-based chemotherapy experienced some degree of hearing loss [23]. In an international collaborative study, we showed that 7.5% of survivors had auditory complications that were associated with a reduced quality of life [24]. Over half of survivors exposed to ototoxic treatments were unaware of their risk of hearing loss, emphasizing the need for improved education and follow-up care [25].
- Nutrition overweight: We found that the dietary intake and quality of survivors was poor, but similar to those of the general population [26, 27]. Survivors were similarly at risk of being overweight to their peers and we identified socio-demographic factors and lifestyle, rather than treatments, to be key drivers of overweight and obesity [28–30].
- Musculoskeletal late effects: We found a cumulative incidence of musculoskeletal late effects of 26% and these conditions were increasingly observed in more recently treated participants [31].

SCCSS data contributed also to international studies coordinated by the Pan-European Network for Care of Survivors after Childhood and Adolescent Cancer (PanCare):

PanCareSurFup (PanCare childhood and adolescent cancer survivor care and follow-up studies; http://www.pancaresurfup.eu/), where the SCCSS contributed with 4719 Swiss 5-year survivors in studies on late mortality [32], second cancers [33, 34], and cardiac late effects [35, 36].

PanCareLIFE (PanCare Studies in Fertility and Ototoxicity to Improve Quality of Life after Cancer during Childhood, Adolescence and Young Adulthood; http://www.pancarelife.eu/), where the SCCSS contributed to work packages on hearing loss and HRQoL, with publications ongoing [24, 37, 38].

## What are the main strengths and weaknesses?

The SCCSS has several strengths, some of which distinguish it from other survivor cohorts:

- Its national, population-based design and direct linkage to the ChCR ensures representativeness for all Swiss survivors diagnosed since 1976.
- Being a dynamic cohort, the continuous recruitment of new 5-year survivors enables the investigation of time trends and outcomes related to evolving treatment protocols.
- The inclusion of survivors during childhood and adolescence—uncommon in similar cohorts—enables the investigation of early outcomes.
- Methodological alignment and harmonization of core questionnaire items with international studies (e.g. SCCSS) support data pooling.
- Outcome data that are independent of participation in questionnaire surveys or nested studies, such as mortality and second primary neoplasms, are available for all registered survivors, minimizing selection bias.
- A sibling control group and national reference data (Supplementary Tables 1 and 3) allow comparisons with populations unaffected by cancer.
- Strong collaboration with pediatric oncology centers facilitates nested studies and the integration of routine hospital data.
- Linked biobanks allow research on projects targeting personalized medicine, gene–treatment interactions, and translational research.
- Active patient and family involvement with regular communication (newsletters, social media, stakeholder meetings) and collaboration ensures relevance for affected patients and their families.

The SCCSS also faces limitations:

- Objective clinical assessments are currently limited to selected outcomes (e.g. cardiac, pulmonary, hearing) and are available only for subsets of the cohort in specific hospitals.
- Although the questionnaire response rates remain high, they have declined slightly over time, potentially affecting generalizability. However, analyses suggest limited bias [39].
- Lower participation during follow-up may have introduced attrition bias if respondents differed systematically from those lost to follow-up.
- The rarity of childhood cancers limits statistical power for rare outcomes and exposures, though this is partly mitigated through international collaborations.
- Detailed treatment data, including dosage information, are only available for a subset of survivors. Retrospective collection is resource-intensive but expected to improve in the future with the electronic capturing of drug administration within pediatric hospitals.
- Germ-line DNA and sequencing data and tumor samples are not yet available for all.
- As baseline surveys begin 5 years post-diagnosis, information on the early survivorship period remains limited.

## Can I get hold of the data? Where can I find out more?

The SCCSS has a policy of collaborative research and is open for data sharing. Data can be made available for researchers who fulfill all of the legal requirements; detailed information is available on the SCCSS website at www.swiss-ccss.ch. Requests for access to the data or collaboration can be addressed to https://www.childhoodcancerregistry.ch/ for ChCR data, to https://cansearch.ch/en/2-biobank-infrastructure-bahop-biskids-biolink/ for germ-line data, and to https://www.kispi.uzh.ch/forschungszentrum/forschungsgebiete/onkologie-fzk for tumor data.

## Supplementary Material

dyag089_Supplementary_Data

## Data Availability

The data that support the information of this manuscript were accessed on secured servers of the ISPM at the University of Bern. Individual-level, fully anonymized, sensitive data can only be made available for researchers who fulfill the respective legal requirements. Requests for data from the Childhood Cancer Registry must be directed to the Childhood Cancer Registry of Switzerland (https://www.childhoodcancerregistry.ch). Requests for data from the SCCSS should be communicated to the study lead, Claudia E. Kuehni (claudia.kuehni@unibe.ch).
