# Supplementary material for: Cohort Profile Update: The Swiss Childhood Cancer Survivor Cohort
Source: Int J Epidemiol. 2026 Jun 15;55(3):dyag089. doi: 10.1093/ije/dyag089 (PMC13265378; doi:10.1093/ije/dyag089)
Supplement: dyag089_Supplementary_Data [file dyag089_supplementary_data.docx]

**Supplementary file**

**Table S1**: Siblings who participated in the Swiss Childhood Cancer Survivor Study by 2025: completed questionnaires and response rates

|  | **Sibling participants (response rate)** | | | |
| --- | --- | --- | --- | --- |
|  | Adults  (aged ≥20)  at survey | Adolescents (aged 16-19)  at survey | Children  (aged 5-15)  at survey | **Total** |
| Siblings of Wave 2007-2013 survivors,  Mailing 2010-2013 | 600 (62%) | 141 (46%) | 127 (50%) | **868 (57%)** |
| Siblings of Wave 2015-2017 survivors,  Mailing 2022-2023 | 81 (34%) | 24 (34%) | 28 (38%) | **133 (35%)** |
| Siblings of Wave 2021-2022 survivors,  Mailing 2023-2024 | 82 (41%) | 30 (45%) | 62 (54%) | **174 (46%)** |
| **Total Siblings**  **as of July 31^st^, 2025** | **763 (54%)** | **195 (44%)** | **217 (49%)** | **1175 (51%)** |

**Table S2:** Age distribution of the Swiss Childhood Cancer Survivor Study (SCCSS) cohort and nested studies as of July 31^st^, 2025. This includes all survivors who are still alive and eligible for analyses on late mortality and 2^nd^ primary neoplasms (Column A, N=8036), and those who answered at least one questionnaire (Column B, age when they answered the baseline questionnaire; column C: age as of July 31, 2025). Columns D-H present the age distribution at the time of study baseline of nested studies.

| Age (years) as of July 31, 2025 | **A:** ChCR:  all registered childhood cancer patients who are alive  N (%) ^a^ | **B:** SCCSS: Number of respondents –  age at baseline questionnaire (%) | **C:** SCCSS: Number of respondents – age as of July 31, 2025 | **D:** SCCSS- CardioOnco: Number of respondents – age at study baseline | **E:** SCCSS-Pulmo: Number of respondents – age at study baseline | **F:** SCCSS-HEAR: Number of respondents – age at study baseline | **G:** SCCSS-Activity: Number of respondents – age at study baseline | **H:** SCCSS-Nutrition: Number of respondents – age at study baseline |
| --- | --- | --- | --- | --- | --- | --- | --- | --- |
|  | **8036** | **4131-4139** ^c^ | **4128-4136** ^c^ | **587-595** ^c^ | **251** | **314-322** ^c^ | **127** | **796-804**^c^ |
| 5-9 years | 521 (7%)^b^ | 332 (8%) | 58 (1%) | - | 61 (24%) | - | 20 (16%) | - |
| 10-14 | 734 (9%) ^b^ | 774 (19%) | 214 (5%) | - | 91 (36%) | - | 37 (29%) | - |
| 15-24 | 2142 (27%) ^b^ | 1729 (42%) | 870 (21%) | 156 (26-27%) ^c^ | 99 (40%) | 66 (20-21%) ^c^ | 44 (35%) | 69 (9%) |
| 25-34 | 2061 (26%) | 897 (22%) | 1138 (28%) | 210 (35-36%) ^c^ | - | 111 (34-35%) ^c^ | 26 (20%) | 343 (43%) |
| 35-44 | 1477 (18%) | 323 (8%) | 1056 (26%) | 160 (27%) | - | 92 (29%) | - | 279 (35%) |
| 45-54 | 820 (10%) | 76 (2%) | 603 (15%) | 61 (10%) | - | 45 (14%) | - | 105 (13%) |
| 55-64 | 269 (3%) | <10 (<1%) ^c^ | 189 (5%) | <10 (<2%) ^c^ | - | <10 (<3%) ^c^ | - | <10 (<2%) ^c^ |
| 65+ | 12 (<1%) | - | <10 (<1%) ^c^ | - | - | - | - | - |

^a^ We considered all ChCR participants with a coded and validated cancer diagnosis in the registry according to the International Classification of Childhood Cancer, 3rd edition (ICCC-3). Survivors diagnosed from 2024 have not been coded and validated yet. ^b^ Among these three age groups, not all have reached 5 years since diagnosis, so only some of them have been eligible for questionnaire surveys. ^c^ We suppressed categories with <10 to protect participant anonymity. We present the total N and corresponding percentages as ranges to prevent the back-calculation of these suppressed values. Percentages may not total 100% due to rounding.

**Table S3: Datasets used or anticipated for comparison in the Swiss Childhood Cancer Survivor Study (SCCSS)**

| **Study** | **Study description** |  |
| --- | --- | --- |
| **Comparison groups with the general population** | |  |
| Swiss Health Survey (SHS) ^1^ | The Swiss Health Survey (SHS) is a nationally representative telephone and questionnaire survey that has been conducted every five years since 1992 by the Federal Statistical Office. The survey includes questions about lifestyle, physical and mental health, health service utilization, and socio-demographic information. The target population includes all individuals aged 15 and older living in private households in Switzerland. In the for the SCCSS relevant time frame (since 2006), a total of 84 421 individuals participated. |  |
| Swiss Health and Lifestyle Survey ^2^ | The Health and Lifestyle survey is a representative online survey with Swiss residents aged 15 years and older on (alternative) tobacco and nicotine consumption, other drugs and behavioral addictions, such as cannabis and medication, and health behaviors, such as physical activity. Since its inception in 2018, 22 995 individuals participated. |  |
| Swiss Multicenter Adolescent Survey on Health (SMASH) ^3^ | The SMASH study was a national, representative school-based questionnaire study with adolescents aged 16 to 20 years. The questionnaire inquired about lifestyle, physical and social environment, physical and emotional health, and medical care. SMASH collected data in 1992/1993 and in 2002 ^4^, including a total of 16 696 adolescents. |  |
| Health Behavior in School Aged Children (HBSC) ^5^ | The HBSC study was initiated in 1982 as a large international questionnaire study on health behaviors of adolescents (11-15 years) in the general population, with data being collected every four years. Switzerland is one of the participating countries, recruiting a nationally representative sample. In the for the SCCSS relevant time frame (since 2006), a total of 32 172 adolescents participated ^6^. |  |
| Motorik-Modul (MoMo) Longitudinal Study ^7^ | The MoMo-Study is a nationally representative study on physical activity, physical fitness, and health among 4–17-year-old children in Germany, including both repeated cross-sectional and longitudinal questionnaire, anthropometric, and physical fitness assessments. The questionnaire inquires about physical activity, physical and mental health, health behaviors, and individual, social, and environmental factors. Since its inception in 2003, the study has recruited 20 012 individuals. |  |
| **Comparison datasets with childhood cancer survivors** | | |
| Childhood Cancer Survivor Study (CCSS) ^8^ | | CCSS is a long-term follow-up cohort study of 31 institutions in the United States and Canada of 5-year survivors diagnosed since 1970 with one of the following: leukemia, CNS cancers, Hodgkin’s or non-Hodgkin’s lymphoma, Wilms (kidney) tumor, neuroblastoma, soft tissue sarcoma, or bone tumor ^8^. The study was initiated in 1994 and utilizes a comprehensive baseline questionnaire, eight follow-up and multiple ancillary study questionnaires to date ^9^. As of January 2021, the entry and expansion cohort include a total of 25 735 survivors ^10^. For comparison, siblings of a subset of randomly selected survivors were also included in the study. As of January 2021, 5045 siblings are part of the cohort ^10^. |
| St. Jude Lifetime Cohort Study (SJLIFE) ^11^ | | SJLIFE is a long-term follow-up study of childhood cancer survivors diagnosed with cancer before the age of 25 years who have been treated at St. Jude’s Children Hospital between 1962 and 2012 and who have survived ≥5 years since diagnosis. The study includes a comprehensive health questionnaire and detailed medical assessments. As of October 2022, the SJLIFE Cohort comprises 9366 participants ^12^. SJLIFE recruits age-, sex- and race-matched community individuals as a comparison group. |

**References**

1. Federal Statistical Office FSO. Die Schweizerische Gesundheitsbefragung 2022 in Kürze [The Swiss Health Survey Summarized - Concept, Methods, Implementation], 2023.

2. Federal Office of Public Health FOPH. Health and lifestyle Survey. <https://www.bag.admin.ch/en/health-and-lifestyle-survey> (7 May 2026, date last accessed).

3. Narring F, Michaud PA. Methodological issues in adolescent health surveys: the case of the Swiss Multicenter-adolescent Survey on Health. *Soz Praventivmed* 1995; **40**(3): 172–82. doi:10.1007/BF01318638

4. Jeannin A, Narring F, Tschumper A, et al. Self-reported health needs and use of primary health care services by adolescents enrolled in post-mandatory schools or vocational training programmes in Switzerland. *Swiss Medical Weekly* 2005; **135**(1-2): 11–8.

5. Roberts C, Currie C, Samdal O, Currie D, Smith R, Maes L. Measuring the health and health behaviours of adolescents through cross-national survey research: recent developments in the Health Behaviour in School-aged Children (HBSC) study. *Journal of Public Health* 2007; **15**(3): 179–86. doi:10.1007/s10389-007-0100-x

6. Balsiger N, Delgrande Jordan M. Gesundheit und Gesundheitsverhalten von 11-, 13- und 15-jährigen Jugendlichen im Jahr 2022 und Entwicklung über die Zeit: Die Schweiz im internationalen Vergleich - Ergebnisse der Studie Health Behavior in School-aged Children (HBSC) (Forschungsbericht Nr. 176). [Health and health behaviors of 11-, 13-, and 15-year old adolescents in 2022 and time trends: Switzerland and international comparison - Results of the study Health Behavior in School-Aged Children (HBSC)]. Lausanne: Sucht Schweiz, 2025.

7. Woll A, Klos L, Burchartz A, et al. Cohort Profile Update: The Motorik-Modul (MoMo) Longitudinal Study-physical fitness and physical activity as determinants of health development in German children and adolescents. *Int J Epidemiol* 2021; **50**(2): 393–4. doi:10.1093/ije/dyaa281

8. Robison LL, Armstrong GT, Boice JD, et al. The Childhood Cancer Survivor Study: a National Cancer Institute-supported resource for outcome and intervention research. *J Clin Oncol* 2009; **27**(14): 2308–18. doi:10.1200/JCO.2009.22.3339

9. Childhood Cancer Survivor Study. Cohort Study Questionnaires and Data Dictionaries. <https://ccss.stjude.org/resources/questionnaires.html> (7 May 2026, date last accessed).

10. Childhood Cancer Survivor Study. Public Access Data Tables. Overall CCSS Cohort Demographic and Treatment Exposure Tables. <https://ccss.stjude.org/public-access-data/tables.html> (7 May 2026, date last accessed).

11. Howell CR, Bjornard KL, Ness KK, et al. Cohort Profile: The St. Jude Lifetime Cohort Study (SJLIFE) for paediatric cancer survivors. *Int J Epidemiol* 2021; **50**(1): 39–49. doi:10.1093/ije/dyaa203

12. St. Jude Children's Research Hospital. St. Jude Lifetime Cohort Study. Study Population. <https://sjlife.stjude.org/investigators/study-population.html> (7 May 2026, date last accessed).
